# Clinical and genetic characteristics of Chinese patients with familial or sporadic pediatric cataract

**DOI:** 10.1186/s13023-018-0828-0

**Published:** 2018-06-18

**Authors:** Jingyan Li, Yunji Leng, Shirui Han, Lulu Yan, Chaoxia Lu, Yang Luo, Xue Zhang, Lihua Cao

**Affiliations:** 1 The Research Center for Medical Genomics, Key Laboratory of Cell Biology, Ministry of Public Health, Key Laboratory of Medical Cell Biology, Ministry of Education, College of Basic Medical Science, China Medical University, No.77 Puhe Road, Shenyang North New Area, Shenyang, 110122 China; 2 McKusick-Zhang Center for Genetic Medicine, State Key Laboratory of Medical Molecular Biology, Institute of Basic Medical Sciences, Chinese Academy of Medical Sciences and Peking Union Medical College, Beijing, 100005 China

**Keywords:** Pediatric cataract, Next-generation sequencing, Variant, Nystagmus

## Abstract

**Background:**

Pediatric cataract is a clinically and genetically heterogeneous disease which is a significant cause of lifelong visual impairment and treatable blindness. Our study aims to investigate the genotype spectrum in a group of Chinese patients with pediatric cataract.

**Methods:**

We enrolled 39 families with pediatric cataract from October 2015 to April 2016. DNA samples of the probands were analyzed by target next-generation sequencing. Variants were validated using Sanger sequencing in the probands and available family members.

**Results:**

In our cohort of 39 cases with different types of pediatric cataract, 23 cases were found to harbor putative pathogenic variants in 15 genes: *CRYAA*, *CRYBA1*, *CRYBA4*, *CRYBB1*, *CRYGC*, *CRYGD*, *MIP*, *GCNT2*, *IARS2*, *NHS*, *BCOR*, *BFSP2*, *FYCO1*, *MAF*, and *PAX6*. The mutation detection rates in the familial and sporadic cases were 75 and 47.8%, respectively. Of the 23 causative variants, over half were novel.

**Conclusions:**

This is a rare report of systematic mutation screening analysis of pediatric cataract in a comparably large cohort of Chinese patients. Our observations enrich the mutation spectrum of pediatric cataract. Next-generation sequencing provides significant diagnostic information for pediatric cataract cases, especially when considering sporadic and subtle syndromal cases.

**Electronic supplementary material:**

The online version of this article (10.1186/s13023-018-0828-0) contains supplementary material, which is available to authorized users.

## Background

Pediatric cataract is often referred to as congenital or infantile cataract, characterized by any opacity of the lens presenting at birth or in the first year of life. With a global prevalence of 3–6 in 10,000 live births and accounting for 10% of childhood blindness worldwide, pediatric cataract is one of the most common causes of visual impairment and blindness in children [1–3]. Pediatric cataract either occurs as a systemic (syndromic) disease or as an isolated (non-syndromic) disease with or without other ocular malformations such as microcornea, microphthalmia, or anterior segment dysgenesis [4, 5]. While autosomal dominant inheritance is most common, autosomal recessive and X-linked inheritance have also been reported, indicating some degree of genetic heterogeneity in pediatric cataract. However, only 8–25% of cases have cataract-linked, inherited mutations [6]. Approximately 81.2% of pediatric cataract cases do not have a family history, suggesting that a significant proportion of cases are sporadic, but many of these cases lack a known underlying genetic cause [7].

Mutations in over 318 genes associated with cataracts had been reported prior to 29 January 2018 (http://cat-map.wustl.edu/), including genes coding for crystallins, intermediate filament proteins, cytoskeleton proteins, gap junction proteins, lens membrane proteins, and lens-associated transcription factors [8]. In this study, we characterize the clinical manifestations and identify pathogenic variants in a cohort of 39 pediatric cataract cases with a variety of inheritance patterns, including a high proportion of sporadic cases in non-consanguineous families. Determining the precise genetic causes of pediatric cataract has significant clinical relevance for defining clinical diagnoses, implementing early treatment strategies, and guiding genetic counseling.

## Methods

### Participants

Thirty-nine probands with bilateral pediatric cataract were investigated as part of this study, including 22 total cataracts, three perinuclear cataracts, two nuclear cataracts, one posterior polar cataract, and 11 undetermined types. All patients with histories of intrauterine infection, drug exposure, metabolic disorders, or malnutrition were excluded. A positive family history was observed in 41.02% (16/39), and non-syndromic cataract was the most common presentation (34/39). A pedigree analysis of the 16 familial cases suggested that 13 were caused by an autosomal dominant mode of inheritance and the remaining three were likely caused by autosomal recessive variants with no consanguinity. While 38 probands were diagnosed within the first year of life, the remaining proband was diagnosed at 5 years old. 15 probands also had nystagmus, six had microphthalmia and/or microcornea, and five had extra-ocular features.

### Panel design, library preparation and next-generation sequencing

A panel of amplicons, targeting the coding exons and 25 bp flanking intronic sequences of 80 cataract-associated genes, was designed by combining data from the Online Mendelian Inheritance in Man (https://omim.org/) and an independent search of PubMed literature (https://www.ncbi.nlm.nih.gov/pubmed). The gene list is shown in Additional file 1: Table S1. The panel included 1811 amplicons with lengths ranging from 125 to 375 base pairs, covering 98.16% of the bases in the target regions.

Libraries were constructed using the Ion AmpliSeq Library Kit v2.0, and DNA fragments from individual samples were ligated with barcoded sequencing adaptors using the Ion Xpress Barcode Adapter 1–16 Kit according to the manufacturer’s instructions. Bar-coded libraries were selectively amplified by emulsion PCR, and ion sphere particles with qualified DNA were isolated and sequenced on Ion 318 Chips using the vendor-provided sequencing kit on the Ion Personal Genome Machine Sequencer (Life Technologies, Carlsbad, CA). Variants were initially called using Ion Torrent Variant Caller version 4.0 software and subsequently visualized using the Integrative Genomics Viewer to facilitate the detection of false variant calls. Confirmed variants were annotated using ANNOVAR (http://wannovar.wglab.org/), and respective minor allele frequencies were assesed in dbSNP (http://www.ncbi.nlm.nih.gov/projects/SNP), 1000genomes (http://www.1000genomes.org/), Exome Variant Server (http://evs.gs.washington.edu/EVS/) and Exome Aggregation Consortium (ExAC) databases (http://exac.broadinstitute.org/). Heterozygous variants with minor allele frequencies > 0.01 were filtered out. Variants were validated using Sanger sequencing in the probands and available family members, and then analyzed for possible pathogenic significance according to the 2015 American College of Medical Genetics and Genomics (ACMG) guidelines [9].

### Haplotype analysis and allele specific PCR

Six short tandem repeat (STR) microsatellite markers flanking *PAX6* were genotyped in family #12, and six STR markers flanking *GCNT2* were genotyped in family #9 and sporadic case #5. PCR products were separated by electrophoresis on 8% denaturing polyacrylamide gel, and allele fragments were detected with routine silver staining. Haplotypes were determined based on each individual’s genotype and kinship. To examine the low-level mosaicism in the unaffected parents in family #12, allele-specific PCR was performed with primers designed for the mutant allele. PCR products were detected by agarose gel electrophoresis. The primer sequences are listed in Additional file 2: Table S2.

## Results

### Targeted region analysis

Next-generation sequencing (NGS) was performed on DNA from the 39 perdiatric cataract probands to detect variants. NGS yielded an output of 5.38G bases with an average of 5.3 M reads/ chip. At least 230,000 reads with a quality score of AQ20 were obtained per sample, with a coverage of approximately 98.15% for the targeted regions and an average depth of 160. The mean read length was 211 bp (Additional file 3: Table S3).

### Identification of suspected causative variants

**Fig. 1:**
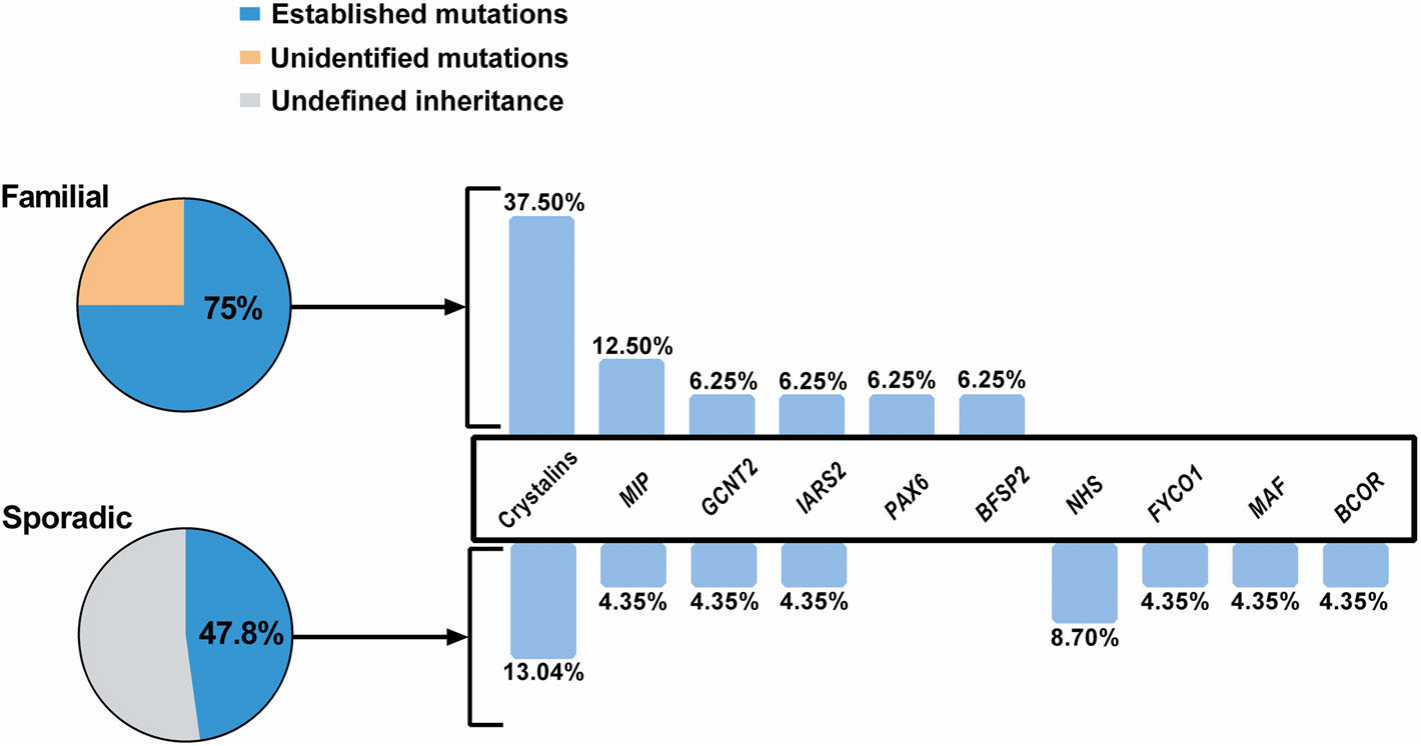
Mutation spectrum of familial and sporadic pediatric cataract cases. The mutation detection rates in the familial and sporadic cases were 75 and 47.8%, respectively. Mutations were found in 15 different genes, with high-penetrance mutations distributed in crystallins, *MIP*, *GCNT2*, *IARS2*, and *NHS*

Twenty-three of the 39 cases tested harbored putative pathogenic variants (Table 1), with mutation detection rates in the familial and sporadic cases of 75% (12/16) and 47.8% (11/23) (Fig. 1), respectively. These variants were spread over 15 cataract-associated genes, with variations in crystallins (*CRYAA*, *CRYBA1*, *CRYBA4*, *CRYBB1*, *CRYGC*, *CRYGD*) accounting for 39.13% (9/23) of the cases. In addition, likely causative variants were found in *MIP* in three families; *GCNT2*, *IARS2*, and *NHS* in two families each; and *BCOR*, *BFSP2*, *FYCO1*, *MAF*, and *PAX6* in one family each. Among the 23 causative variants identified in this study, 12 variants were novel, with the remaining 11 variants already reported. According to the ACMG mutation guidelines, all of the variants were classified as “pathogenic” or “likely pathogenic”. Additionally, four variants classified as “uncertain significance” were identified in two familial and two sporadic cases (Additional file 4: Table S4 and Additional file 5: Figure S1). Two familial cases and 10 individuals with sporadic pediatric cataract had no variants of interest found in the 80 cataract-associated genes screened through this study (Additional file 5: Figure S1).

**Table 1:** Familial and sporadic pediatric cataracts with likely causative variants

Proband ID	Sex	Age of onset	Ocular phenotype	Extraocular features	Inheritance,before and after testing	Gene Refseq ID	Nucleotide change	Predicted amino acid change	Protein domain	SIFT/MutTaster/PolyPhen	MAF (ExAC, East Asian)	ACMG	Novel
F#1	F	1 year	Perinuclear cararact, microphthalmia	-	AD	*CRYAA NM_000394.3*	c.61C>T	p.(Arg21Trp)	Alpha-crystallin N-terminal	D, D, D	-	P	Ref [35]
F#2	M	Birth	Total cataract	-	AD	*CRYBA1 NM_005208.4*	c.552_557delinsGGAGG	p.(Cys185Glufs*33)	-	-	-	P	Yes
F#3	F	Birth	Total cataract, microphthalmia	-	AD	*CRYBA4 NM_001886.2*	c.277T>C	p.(Ser93Pro)	2nd Greek key	D, D, D	-	P	Yes
S#1	F	Birth	Total cataract, nystagmus	-	Sporadic->new AD	*CRYBB1 NM_001887.3*	c.508G>T	p.(Asp170Tyr)	3rd Greek key	D, D, D	-	P	Yes
F#4	M	5 years	Nuclear cataract	-	AD	*CRYGC NM_020989.3*	c.233C>T	p.(Ser78Phe)	2nd Greek key	D, D, D	-	LP	Yes
F#5	M	Birth	Unknown type	-	AD	*CRYGD NM_006891.3*	c.70C>A	p.(Pro24Thr)	1st Greek key	T, D, B	-	P	Ref [10, 11]
S#2	M	Birth	Unknown type		Sporadic->new AD	*CRYGD NM_006891.3*	c.134T>C	p.(Leu45Pro)	2nd Greek key	D, D, D	-	P	Ref [12]
F#6	F	Birth	Total cataract, strabismus, nystagmus	-	AD	*CRYGD NM_006891.3*	c.309dup	p.(Glu104Argfs*4)	-	-	-	P	Ref [36]
S#3	F	Birth	Total cataract, nystagmus	-	Sporadic->new AD	*CRYGD NM_006891.3*	c.418C>T	p.(Arg140*)	-	-	-	P	Ref [37]
F#7	F	2 months	Unknown type, nystagmus	-	AD	*MIP NM_012064.3*	c.494G>A	p.(Gly165Asp)	Aquaporin-like	D, D, D	-	P	Ref [17]
S#4	F	3 months	Total cataract, nystagmus	-	Sporadic->new AD	*MIP NM_012064.3*	c.530A>G	p.(Tyr177Cys)	Aquaporin-like	T, D, D	-	P	Ref [18]
F#8	F	Birth	Unknown type, nystagmus	-	AD	*MIP NM_012064.3*	c.612C>G	p.(Tyr204*)	-	-	-	P	Yes
S#5/F#9	M/F	3/5 months	Total cataract, nystagmus/Total cataract	-	Sporadic/AR->AR	*GCNT2 NM_001491.2*	c.1043G>A	p.(Gly348Glu)	Lumenal domain	D, D, D	0.00104	P	Ref [15]
							c.1148G>A	p.(Arg383His)	Lumenal domain	D, D, B	0.0003467	P	
S#6	M	5 months	Perinuclear cararact	-	Sporadic->AR	*IARS2 NM_018060.3*	c.607G>C	p.(Gly203Arg)	Aminoacyl-tRNA synthetase	D, D, D		LP	Yes
							c.2575T>C	p.(Phe859Leu)	Anticodon-binding	T, D, D	0.002775	LP	
F#10	M	6 months	Unknown type	-	AR	*IARS2 NM_018060.3*	c.2446C>T	p.(Arg816*)	-	-	0.0001156	P	Yes
							c.2575T>C	p.(Phe859Leu)	Anticodon-binding	T, D, D	0.002775	LP	
S#7	M	Birth	Unknown type, microphthalmia, microcornea, nystagmus	Long narrow face, small nose, mild anteverted pinnae, and dental anomalies	Sporadic->X-linked	*NHS NM_001291868.1*	c.2739del	p.(Phe913Leufs*9)	-	-	-	P	Yes
S#8	M	Birth	Total cataract, microphthalmia, microcornea, nystagmus	Large anteverted pinnae, mental retardation	Sporadic/new X-linked	*NHS NM_001291868.1*	c.3207_3208del	p.(Ala1070Phefs*16)	-	-	-	P	Yes
S#9	F	Birth	Total cataract, microphthalmia, microcornea	Hypodontia	Sporadic->new X-linked	*BCOR NM_001123384.1*	c.4706dup	p.(Gly1570Argfs*7)	-	-	-	P	No
F#11	F	Birth	Total cataract	-	AD	*BFSP2 NM_003571.3*	c.697_699del	p.(Glu233del)	Intermediate filament rod domain	-	-	p	Ref [13]
S#10	M	7 months	Unknown type	-	Sporadic->AR	*FYCO1 NM_024513.3*	c.808C>T	p.(Gln270*)	-	-	-	P	Yes
							c.3587+1G>T		-	-	0.0002326	P	
S#11	M	Birth	Posterior polar cataract	-	Sporadic->new AD	*MAF NM_001031804.2*	c.950A>G	p.(Glu317Gly)	b-Zipper	D, D, D	-	P	Yes
F#12	M	3 months	Unknown type, nystagmus	-	AR->AD	*PAX6 NM_001310159.1*	c.113G>A	p.(Arg38Gln)	Paired domain	D, -, D	-	P	Yes

Abbreviations: Proband ID, *F* family, *S* sporadic case, Sex, *F* female, *M* male; D, damaging, *T* tolerated, *B* benign, *ACMG* American College of Medical Genetics and Genomics, *P* pathogenic, *LP* likely pathogenic, *Ref* Reference

### Variants in crystallin genes

**Fig. 2:**
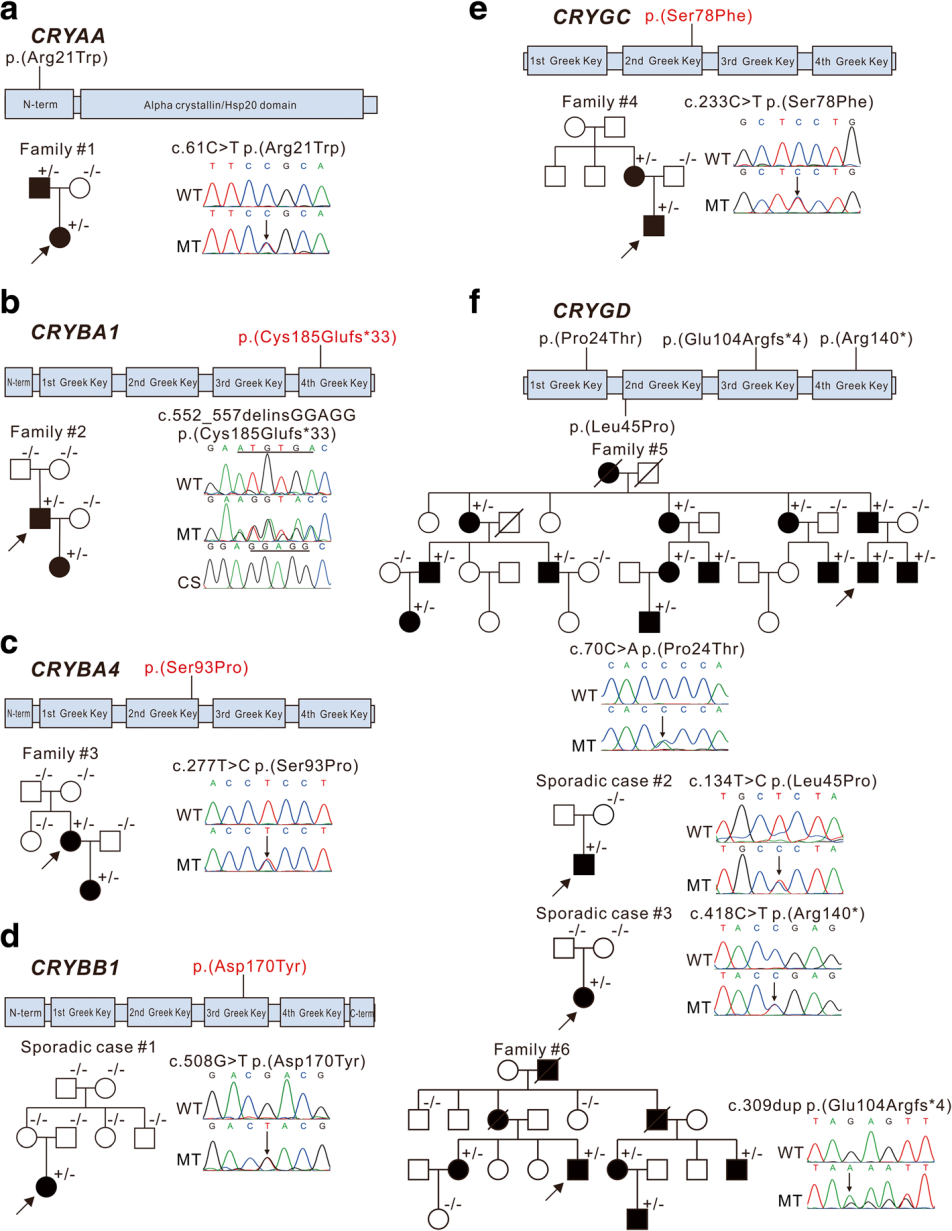
Pedigree and variants identified in crystallin genes These schematics show the encoded domain structure of *CRYAA* (**a**), *CRYBA1* (**b**), *CRYBA4* (**c**), *CRYBB1* (**d**), *CRYGC* (**e**), and *CRYGD* (**f**). Mutations found in this study are illustrated above the schematics, with novel variants indicated in red characters. Probands are indicated by arrows, ^+/−^ indicates heterozygous individuals, ^−/−^ indicates individuals testing negative. WT: wild type, MT: mutant type

Variants in the crystallin genes were the most frequent mutations found in this study, with nine patients presenting likely causative variants in crystallin genes, six in familial cases, and three in sporadic cases. All respective familial cases were caused by autosomal dominant mutations, with the results suggesting that the sporadic cases are new cases of autosomal dominant inheritance. Six of these were caused by missense mutations, two from frameshifts, and one from a nonsense mutation (Fig. 2). Additionally, four of the nine variants were novel: *CRYBA1* c.552_557delinsGGAGG; p.(Cys185Glufs*33), *CRYBA4* c.277 T > C; p.(Ser93Pro), *CRYBB1* c.508G > T; p.(Asp170Tyr), and *CRYGC* c.233C > T; p.(Ser78Phe). All novel missense mutations occurred within a Greek Key motif and might impact on protein folding. The novel heterozygous deletion and insertion in *CRYBA1* (c.552_557delinsGGAGG; p.(Cys185Glufs*33)) is predicted to lead to a premature stop codon, deleting three-fifths of the fourth Greek Key and all of the C-terminal domain of CRYBA3/A1. Five variations of crystallin (*CRYBA1* c.552_557delinsGGAGG; p.(Cys185Glufs*33), *CRYBA4* c.277 T > C; p.(Ser93Pro), *CRYBB1* c.508G > T; p.(Asp170Tyr), *CRYGD* c.309dup; p.(Glu104Argfs*4), and *CRYGD* c.418C > T; p.(Arg140*)) caused total cataract with or without microphthalmia and nystagmus. *CRYGC* c.233C > T; p.(Ser78Phe) caused nuclear cataracts, and *CRYAA* c.61C > T; p.(Arg21Trp) produced perinuclear cataracts and microphthalmia. A hotspot mutation c.70C > A; p.(Pro24Thr) and a previously reported mutation c.134 T > C; p.(Leu45Pro) in *CRYGD* were identified in family #5 and sporadic case #2, with no phenotypic information available [10–12].

### Variants in transcription factor genes *MAF* and *PAX6*

**Fig. 3:**
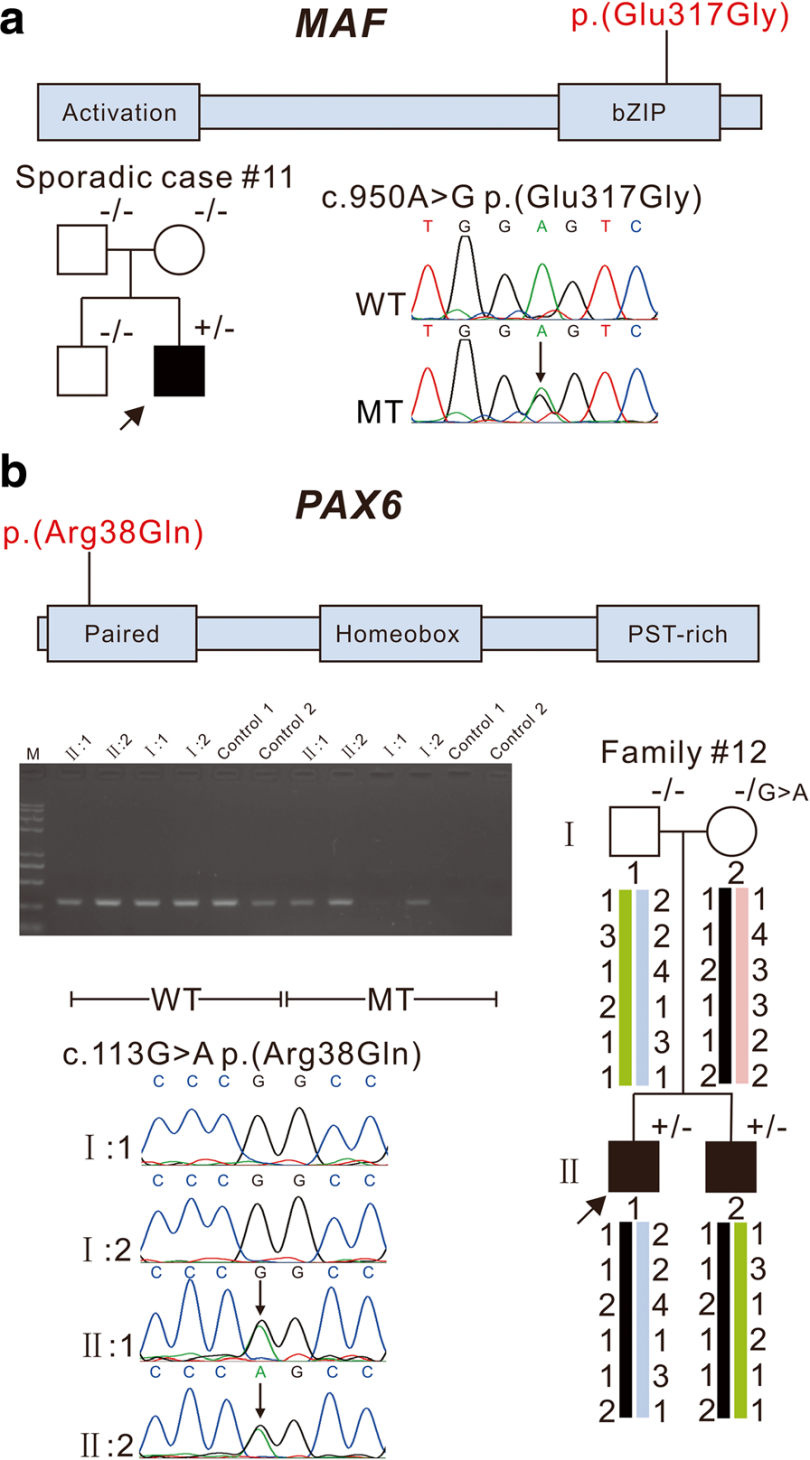
Pedigree and variants in transcription factor genes *MAF* and *PAX6.* The schematics show the encoded domain structure of *MAF* or *PAX6*, and the variants are illustrated above the schematics. *MAF* c.950A > G; p.(Glu317Gly) was identified in sporadic case #11 (**a**). *PAX6* c.113G > A p.(Arg38Gln) was identified in family #12 (**b**). Patients II1 and II2 from family #12 inherited the same *PAX6* allele from their unaffected mother. Allele specific PCR demonstrated that the variant was present in the asymptomatic mother. Probands are indicated by arrows. ^+/−^ indicates heterozygous individuals, ^−/−^ for individuals testing negative, ^−/G > A^ indicates a mosaic case besides the normal sequence “G” also chromosomes are found containing “A”. WT: Wild Type, MT: Mutant Type

A likely de novo novel heterozygous missense mutation c. 950A > G; p.(Glu317Gly) in the bZIP domain of *MAF* was identified in the sporadic case #11(Fig. 3a) who was diagnosed with bilateral posterior polar cataracts.

Another novel variant, c.113G > A; p.(Arg38Gln), in the paired domain of *PAX6,* was found in both the proband of family #12 and his affected brother with cataract and nystagmus, but this variant was not observed via Sanger sequencing in either normal parent. The haplotype analysis demonstrated that both siblings inherited the same *PAX6* allele from their mother, indicating that their mother may be gonadal mosaic for the disorder, and allele specific PCR confirmed that the variant was indeed present in the asymptomatic mother (Fig. 3b).

### Variants in non-syndromal cataract genes *BFSP2*, *FYCO1*, *GCNT2*, and *MIP*

**Fig. 4:**
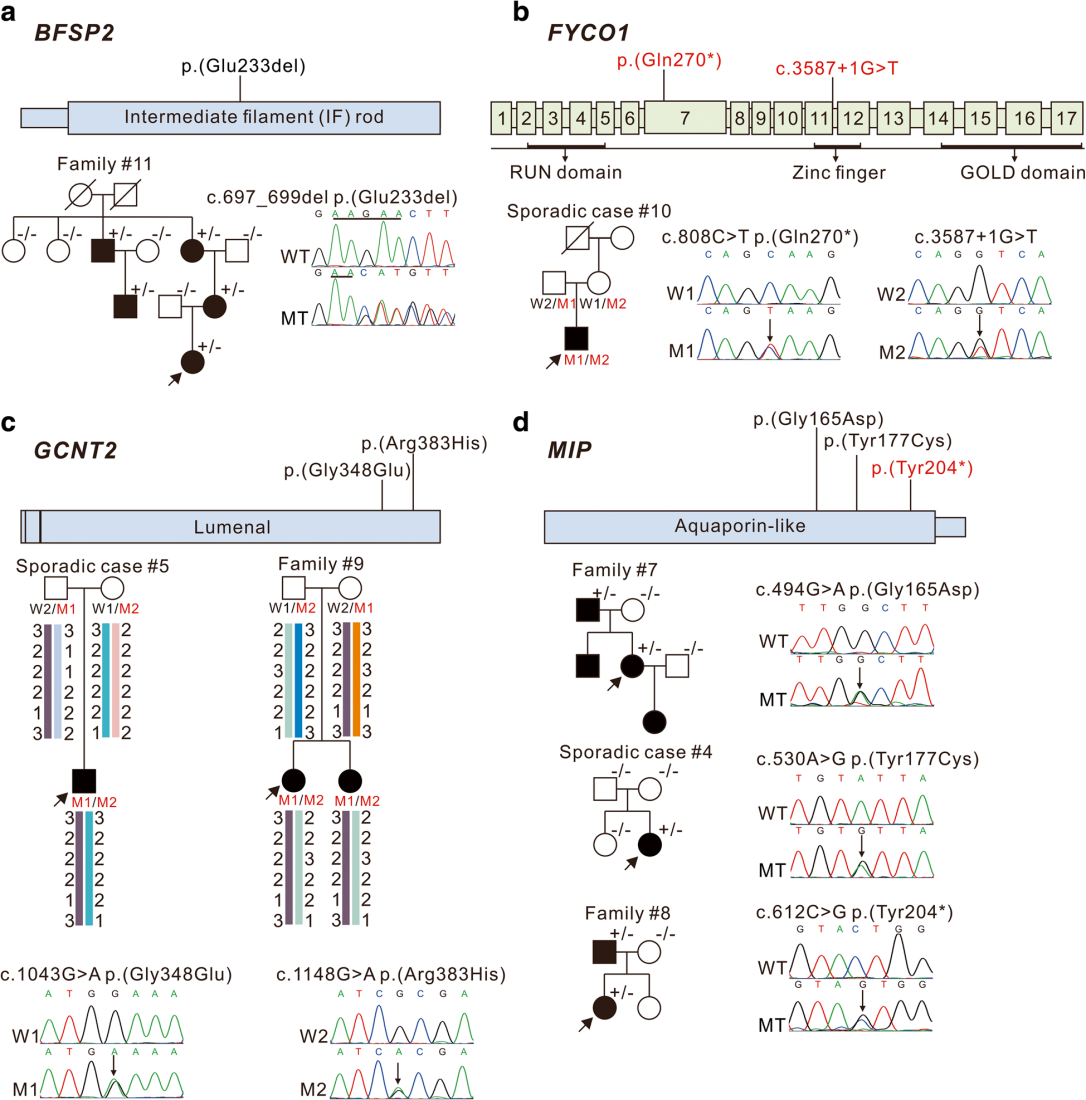
Pedigree and variants in *BFSP2*, *FYCO1*, *GCNT2*, and *MIP.* These schematics show the encoded domain structure of *BFSP2* (**a**), exonic and protein domain structure of *FYCO1* (**b**), encoded protein domain structure of *GCNT2* (**c**) and *MIP* (**d**). Mutations found in this study are illustrated above the schematics, with novel variants indicated in red characters. Probands are indicated by arrows, ^+/−^ indicates heterozygous individuals, ^−/−^ for individuals testing negative. WT: wild type, MT: mutant type, W1: Wild Type 1, W2: Wild Type 2, M1: Mutant Type 1, M2: Mutant Type 2

The *BFSP2* gene encodes phakinin, a lens-specific intermediate filament-like protein. An in-frame deletion c.697_699del; p.(Glu233del) in the intermediate filament rod domain of *BFSP2*, previously reported by Jakobs PM and Zhang Q [13, 14], was identified in all affected individuals of family #11 with total cataract (Fig. 4a).

Novel compound heterozygous variants c. 808C > T; p.(Gln270^*^) and c.3587 + 1G > T in *FYCO1* were identified in the sporadic case #10, with parental segregation subsequently confirmed (Fig. 4b). The nonsense mutation c. 808C > T; p.(Gln270^*^) was predicted to truncate most of the coiled-coil region, as well as the entire FYVE zinc-finger and GOLD domain. Additionally, the G-to-T transversion located in the conserved intron 12 donor splice site (c.3587 + 1G > T) might affect splicing.

Homozygous or compound heterozygous mutations in *GCNT2* caused cataract associated with the rare adult i blood group [15, 16]. The recurrent compound heterozygous mutations c.1043G > A; p.(Gly348Glu) and c.1148G > A; p.(Arg383His) in *GCNT2*, previously reported by Yu [15], were found in two patients from family #9 and sporadic case #5, and parental segregation was subsequently confirmed. Haplotype analysis revealed that the c.1043A allele of family #9 and sporadic case #5 was likely due to a founder effect, and the c.1148A allele origins of these two families were independent (Fig. 4c). I/i blood group typing was not performed since we had no access to fresh blood from the patients.

*MIP* is a less frequently investigated cataract-associated gene, but likely causative variants in *MIP* were identified in three patients, accounting for 13.04% (3/23) of the cases in this study. Two variants were familial and the third was a sporadic case of pediatric cataract. While the two missenses mutations, c.530A > G; p.(Tyr177Cys) and c.494G > A; p.(Gly165Asp), have been previously described [17, 18], the nonsense mutation c.612C > G; p.(Tyr204^*^) in family #8 is a novel variant (Fig. 4d), it might prevent MIP protein transport and reduce the formation of available water channels as well as p.(Lys228Glufs*4), which recently reported by Long X [19]. The cataract types of the patients harboring c.494G > A; p.(Gly165Asp) and c.612C > G; p.(Tyr204^*^) were not available, while the patient with mutation c.530A > G; p.(Tyr177Cys) had total cataract. In addition, all patients with *MIP* variants identified in this study had nystagmus.

### Variants in syndromal cataract genes *BCOR*, *IARS2*, and *NHS*

**Fig. 5:**
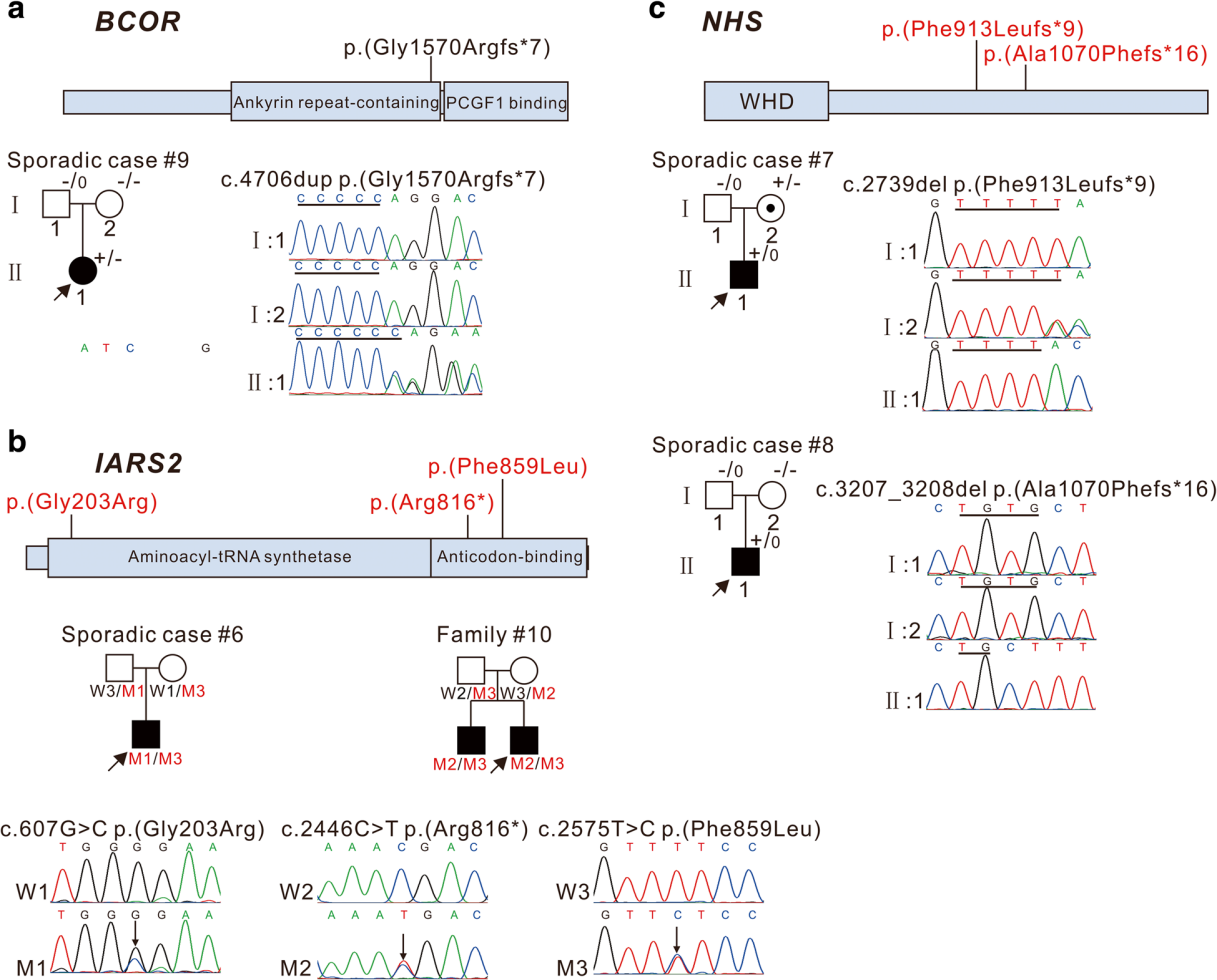
Pedigree and variants in *BCOR*, *IARS2*, and *NHS.* These schematics show the encoded domain structure of *BCOR* (**a**), *IARS2* (**b**), and *NHS* (**c**). *BCOR* and *NHS* are X-linked genes. The variants found in this study are illustrated above the schematics. Probands are indicated by arrows. A dotted circle indicates an obligate X-linked carrier. ^+/−^ indicates heterozygous individual, ^−/−^ indicates individual testing negative, ^+/0^ indicates hemizygote testing positive, ^−/0^ indicates hemizygote testing negative. W1: Wild Type 1, W2: Wild Type 2, W3: Wild Type 3, M1: Mutant Type 1, M2: Mutant Type 2, M3: Mutant Type 3

In sporadic case #9, a likely de novo frameshift mutation c.4706dup; p.(Gly1570Argfs^*^7), was found in *BCOR* (Fig. 5a), the gene responsible for X-linked oculo-facio-cardio-dental (OFCD) syndrome [20]. Although c.4706dup; p.(Gly1570Argfs^*^7) was not reported in the literature, it is included in ClinVar database. The proband had bilateral total cataracts, microphthalmia, and microcornea, with additional dental and facial features consistent with OFCD syndrome. Her mother reported that she tired easily, but she did not undergo any cardiological tests. *BCOR* c.4706dup; p.(Gly1570Argfs^*^7) is predicted to delete part of the Ankyrin repeat-containing domain and the entire PCGF1 binding domain, which is necessary and sufficient for interaction with PCGF1, a component of the Polycomb group (PcG) multiprotein BCOR complex. This interaction is required to maintain the transcriptionally repressive state of BCL6 and CDKN1A [21].

Two novel compound heterozygous mutations in *IARS2*, a nuclear gene encoding mitochondrial isoleucyl-tRNA synthetase [22], were found in family #10 and sporadic case #6 (Fig. 5b). The compound heterozygous variants c.607G > C; p.(Gly203Arg) and c.2575 T > C; p.(Phe859Leu) were identified in the sporadic case #6, who developed a sporadic case of perinuclear cataracts without other anomalies, and the mutations c.2446C > T; p.(Arg816^*^) and c.2575 T > C; p.(Phe859Leu) were identified in two affected brothers in family #10, both of which had bilateral cataracts without other anomalies. p.(Gly203Arg) lies in the class Ia aminoacyl-tRNA synthetases domain, p.(Phe859Leu) localizes to the anticodon-binding domain, and p.(Arg816^*^) would result in a truncated protein lacking the anticodon RNA-binding domain. Segregation studies revealed that both parents were heterozygous for the variants, confirming that these variants were in trans.

Two novel hemizygous frameshift mutations were identified in *NHS*, the gene responsible for X-linked Nance Horan syndrome (NHS) [23, 24]: c.3207_3208del; p. (Ala1070Phefs^*^16) and c.2739del; p.(Phe913Leufs^*^9), in sporadic cases #7 and #8, respectively (Fig. 5c). Both frameshift mutations in *NHS* were predicted to lead to protein truncations. While c.3207_3208del was a likely de novo mutation, c.2739del was heterozygous in the patient’s mother. The sporadic case #7 had bilateral cataracts, microphthalmia, microcornea, and nystagmus, as well as an asymmetric long narrow face, small nose, mild anteverted pinnae, and dental anomalies. His development and intelligence were normal. The sporadic case #8 had bilateral total cataracts, microphthalmia, microcornea, and nystagmus, along with characteristic facial features of a long narrow face, prominent nose, and large anteverted pinnae, dental anomalies of screwdriver-shaped incisors, and an intellectual delay consistent with NHS.

## Discussion

In this study, we used target NGS to identify genetic variants in 39 Chinese probands with inherited pediatric cataract. Twenty-three cases were found to harbor putative pathogenic variants in 15 cataract-associated genes, including missense mutations, nonsense mutations, frameshift deletions/insertions, in-frame deletions, and splicing mutations. All missense mutations identified in this study were located at very important protein domains, and the frameshift deletions/insertions and nonsense mutations found resulted in premature termination codons or triggered a nonsense-mediated mRNA decay. The splicing mutation *FYCO1* c.3587 + 1G > T changed the acceptor site of intron 12, which would generally cause exon skipping. Further functional studies are warranted to determine the physiological implications of each new mutation. The majority of these mutations appear to be autosomal dominant (15/23), with autosomal recessive (5/23) and X-linked changes (3/23) also detected. The most commonly mutated genes were those coding for crystalline, accounting for 39.13% of cases. *MIP* was mutated in three cases, representing the second most commonly mutated gene in our cohort. Interestingly, no gap junction protein-encoding genes were identified in our cohort, although they are frequently reported in non-syndromic pediatric cataract [25, 26].

The large number of genes known to cause pediatric cataract and the limited genotype-phenotype correlations complicate clinical testing using traditional sequencing technologies. These difficulties are especially evident in sporadic pediatric cataract cases, which make up the majority of pediatric cataract cases, and present diagnostic challenges when attempting to identify a genetic etiology [7]. Our study demonstrates that half of the mutations identified in sporadic pediatric cataract cases were due to likely de novo heterozygous mutations in autosomal-dominant genes (5/11), one-fourth were compound heterozygous mutations in autosomal recessive genes (3/11), and one-fourth were X-linked variants (3/11), two of which were likely de novo mutations. Similar difficulties exist in familial cases, as pedigree information alone may not accurately describe the inheritance risk. Family #12 was assumed to have a recessive form of pediatric cataract on the basis of family history, however, genetic testing revealed the presence of a *PAX6* missense mutation in both affected brothers. Since all previously reported mutations in *PAX6* have been dominant [27–29], this finding suggested that their asymptomatic mother was mosaic for the disorder. Moreover, parental mosaicism for mutated *PAX6* in affected siblings has been reported recently [30]. Therefore, the NGS testing results dramatically altered the counseling of both the parents and the patients themselves. Thus, it is useful for parents of affected children, as well as the affected individuals themselves, to use a targeted NGS panel to provide accurate recurrence and transmission risk counseling.

Some syndromal pediatric cataracts may be subtle, with associated systemic features presenting or becoming apparent only in later childhood [8]. The sporadic case #9 carried a novel *BCOR* mutation associated with OFCD syndrome, but she presented with only subtle clinical features, yet her cardiac status should be monitored for signs of disease progression. Mutations in *IARS2* are also commonly associated with syndromal pediatric cataract. While compound heterozygous mutations in *IARS2* were identified in the patients of family #10 and sporadic case #6, these patients did not present with additional anomalies besides the cataract. Thus, growth hormone levels, neurotrophic keratitis, orbital myopathy, and skeletal dysplasia should be monitored through later follow-ups. Other genes, such as *AGK* and *LONP1,* are known to be mutated in syndromic forms of cataract and have also been reported to cause apparently non-syndromic cataract [12, 31]. *IARS2* might be the third example of such genes that can be mutated in both syndromic and non-syndromic forms of pediatric cataract. Future cataract patients with different mutations in *IARS2* will help clarify the phenotypic spectrum.

In recent years, multiple cataract-targeted gene panels have been developed with detection rates of 26–75% [8, 12, 25, 26, 32–34]. We investigated 16 familial and 23 sporadic cases with pediatric cataract in the Chinese population and achieved an overall mutation detection rate of 58.97%, which is almost identical to those reported in similar studies of patients from South Eastern Australia (62%) [25], China (62.96%) [32], and Saudi Arabia(58%) [12], including zero, 7.4 and 23% of sporadic cases, respectively. The mutation detection rate of the familial cases in our study was 75%, comparable to that published recently in familial patients from the UK (75%) [26] and another Australian cohort (73%) [8]. The mutation detection rate for familial cases in our cohort was much higher than that published recently in two studies in Chinese familial patients, with mutation pick-up rates of 50 and 64% [32, 33]. The mutation detection rate for sporadic cases in our study was 47.8%, lower than reported in sporadic patients from Australia (68%) and Saudi Arabia (62.5%) [8, 12]. If the new candidate genes are included, the mutation detection rate in the sporadic patients from Saudi Arabia increases to 75% [12]. A recently published article reported gene mutations screening in sporadic pediatric cataract in a Han Chinese population using target NGS, and identified pathogenic variants in 26% of cases [34], much lower than ours. The relatively low mutation detection rate in Chinese sporadic patients might be due to the number of target genes being different between different panels or different frequencies of mutations occurring different groups. Also, further clinical exome sequencing panels or whole exome sequencing were not performed for patients negative for mutations from the target NGS in our patients or another Chinese cohort.

One advantage in our study is that we obtained DNA samples from the parents of each proband (except sporadic patient #2; the DNA of his father was unavailable) regardless of family history. We also obtained DNA samples from at least two patients in familial cases and performed segregation analysis to confirm the disease-causing variations. Our study also has several limitations. Nearly all patients underwent cataract surgery prior to enrolment in this study, so phenotypic information was determined by reviewing medical records or recalled by the participants or their guardians. Medical records were not available for 11 probands, so the lens phenotype could not be ascertained in detail. The multi-gene panel was designed in October 2015, so cataract genes published after that date were not included. Four variants predicted to have uncertain significance under ACMG guideline were identified in two familial and two sporadic cases, additional studies will be needed to confirm their pathogenicity. In future efforts, clinical exome sequencing panels targeting all OMIM-identified disease genes, or whole exome sequencing analysis might be necessary for mutation negative cases regardless of family history.

## Conclusion

In conclusion, we examined the clinical manifestations and molecular genetic characteristics of 39 Chinese patients with pediatric cataract. Twenty-three putative pathogenic variants were identified, with 12 novel and 11 recurrent. This has led to more accurate genetic diagnoses and recurrence risk counseling, impacting management for each family.

## Additional files

Additional file 1: **Table S1.** List of 80 pediatric cataract genes. (XLS 36 kb)

Additional file 2: **Table S2.** The primers for haplotype analysis and allele specific PCR. (XLS 25 kb)

Additional file 3: **Table S3.** Target NGS analysis. (XLS 28 kb)

Additional file 4: **Table S4.** Pediatric cataract families with uncertain significance variants. (XLS 29 kb)

Additional file 5: **Figure S1.** Pedigrees of the families without suspected causative variants. (TIF 8488 kb)

## Abbreviations

ACMG: American college of medical genetics and genomics
ExAC: Exome aggregation consortium
NGS: Next-generation sequencing
NHS: Nance horan syndrome
OFCD: Oculo-facio-cardio-dental syndrome
PCR: Polymerase chain reaction
STR: Short tandem repeat
